# Trunk Impact Conditions in Mountain Biking: Biomechanical Insights for Back Protector Evaluation

**DOI:** 10.3390/bioengineering13060636

**Published:** 2026-05-29

**Authors:** Sophie Bonte, Arsène Thouzé, Wei Wei, Pierre-Jean Arnoux, Lionel Thollon, Nicolas Bailly

**Affiliations:** 1 Laboratoire de Biomécanique Appliquée, Aix-Marseille Université, Université Gustave Eiffel, 13015 Marseille, France; sophie.bonte@univ-eiffel.fr (S.B.); wei.wei@univ-eiffel.fr (W.W.); pierre-jean.arnoux@univ-eiffel.fr (P.-J.A.); lionel.thollon@univ-eiffel.fr (L.T.); 2 Decathlon SportsLab, 59000 Lille, France; arsene.thouze@decathlon.com

**Keywords:** mountain biking, impact conditions, injury risk, multibody modelling

## Abstract

Background: Mountain biking is increasingly popular but carries a large risk of severe trunk and spinal injuries. However, realistic crash scenarios for back protector design remain poorly characterized. This study aimed to define trunk impact conditions during mountain biking crashes. Methods: A multi-body model for mountain bike accident reconstruction was developed, and its kinematics were validated against real-world crash video footage. The model was then used to assess the influence of initial conditions (speed, slope, crash cause, etc.) on trunk impact kinematics (velocities, forces, pseudo-energy) and spinal loading indicators during forward crashes. Results: Across 288 simulated crashes, the median normal trunk impact velocity (4.61 m/s) and pseudo-energy (48 J) aligned with current test standards, while substantial tangential (5.97 m/s) and rotational (4.90 rad/s) components were also observed. Three main impact types emerged: head–thorax impacts (43.5%), involving a head impact followed by chest impact (Vn: 5.42 m/s, Emax: 59 J); tumbling (25.1%), featuring a head impact followed by back impact (Vn: 3.98 m/s, Emax: 57 J); and overflip–back impacts (20.7%), involving direct back contact (Vn: 3.35 m/s, Emax: 47 J). Conclusion: This study’s results define trunk impact conditions during MTB crashes, informing on realistic boundary conditions for testing and designing back protectors.

## 1. Introduction

Mountain biking (MTB) is a fast-growing sport practiced by millions worldwide [1], exposing riders to challenging terrains, excessive speeds, and technical obstacles. These conditions often lead to falls, resulting in a wide range of injuries (16.8 per 1000 h of exposure [2]), from minor wounds to life-threatening trauma. While upper limb injuries are the most frequent in MTB (40–75% of all emergency cases vs. 5–20% for the trunk [3,4]), the trunk is particularly affected by severe trauma. Nearly 30% of severe injuries (Injury Severity Score ISS > 12) occur in this region (spine, abdomen, or chest), with spinal injuries among those requiring the greatest rate of surgical intervention (56%) [5].

To mitigate these risks, protective gear has been developed to absorb impact energy during crashes. However, the effectiveness of back protectors in sports remains largely unknown because, first, there is no epidemiological data assessing their effectiveness [6,7], and second, MTB back protectors are tested using motorcycle safety standards (EN 1621-2) [8] rather than MTB-specific evaluation. This approach does not account for the unique kinematics of MTB falls, which may involve different impact patterns, speed, and energy transfer mechanisms than those observed in motorcycling. As a result, existing protectors may not effectively reduce injury risk in real-world MTB accidents. Consequently, understanding crash mechanisms and impact conditions is crucial to defining robust boundary conditions for testing and designing protective equipment tailored to the sport’s specific risks.

Among MTB crash types, forward falls, particularly the “Over-the-Bars” (OTB) scenario, are the leading cause of severe injuries [9,10,11]. In such crashes, riders are propelled over the handlebars, mainly after a bad jump landing [9,12] or front-wheel locking due to collision or loss of control [11], that induce both flexion and compression of the spine. One study [12] reported that up to 75% of MTB spinal injuries result from OTB crashes. Despite these statistics, current research has primarily focused on injury epidemiology and limited data exist regarding the precise biomechanics of these falls including impact speeds, angles, and forces applied to the rider’s body. Moreover, these data might be difficult to obtain through experimental reconstructions of MTB crashes or on-terrain data collection because of different challenges such as ethical concerns, the difficulty of replicating realistic fall conditions, and the variability of real-world riding environments. To bridge this gap and provide robust boundary conditions for equipment testing, numerical simulation is very relevant. Multibody (MB) simulation offers a powerful tool for reconstructing crash scenarios and predicting global impact kinematics (like impact velocities, angles, and energies). Compared to experimental methods, it is less time-consuming and easier to implement, allowing for the simulation of hundreds of full-body crash trajectories across varying initial conditions. While MB modelling has been widely used to study head kinematics in urban cycling crashes [13,14], its application to MTB remains limited. Applying this method to MTB crash scenarios could significantly enhance our ability to identify critical impact parameters and optimize protective gear design.

Using multibody modelling, this study aimed to characterize trunk impact conditions across a range of realistic MTB forward crash scenarios. It also investigated the effects of key parameters (riding speed, crash type, and slope) on trunk impacts, in order to inform the evaluation and design of protective equipment.

## 2. Materials and Methods

### 2.1. The Human Model

**Figure 1:**
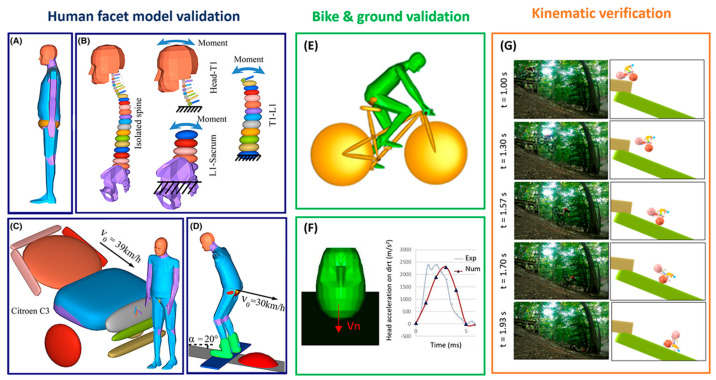
Validation workflow of the mid-size male Human Facet Model (HFM) for MTB accident reconstruction (**A**). HFM validation includes spinal flexion–extension calibration (cervical, thoracic, lumbar) (**B**) and vehicle–pedestrian (**C**) and snowboarding evaluations (**D**). Bike and ground calibration includes multibody bike modelling and HFM coupling (**E**), soil calibration from head–ground impacts based on experimental data from Bland-Rothgeb et al. [16] (**F**), and validation against a real video-based MTB accident (**G**). (**A**–**D**) used with permission of John Wiley & Sons—Books, from Wei et al. [15] permission conveyed through Copyright Clearance Center, Inc.

The human model used to reconstruct the MTB crashes was a Human Facet Model (HFM) representing a 50th percentile male rider (height: 1.74 m, body mass: 75.7 kg) without a helmet (Figure 1A). A key feature of this model is its detailed multibody spine, where each vertebra is defined as an individual rigid body (Figure 1B). Crucially, the biomechanical accuracy and reliability of this specific human model have been rigorously established [15]. Originally developed in MADYMO version R7.5 (TASS International, Delft, The Netherlands), the model was significantly enhanced by Wei et al. (2018) [15]. Its spine flexion–extension range of motion (ROM) was calibrated against the literature data (Figure 1B) and dynamic impact responses were calibrated and validated against cadaveric data and experimental crash reconstructions, including vehicle–pedestrian collisions and backward snowboarding falls (Figure 1C,D). These calibration and validation data are available in the Appendix of Wei et al. (2018) [15].

### 2.2. Mountain Bike Model and Environment

**Figure A1:**
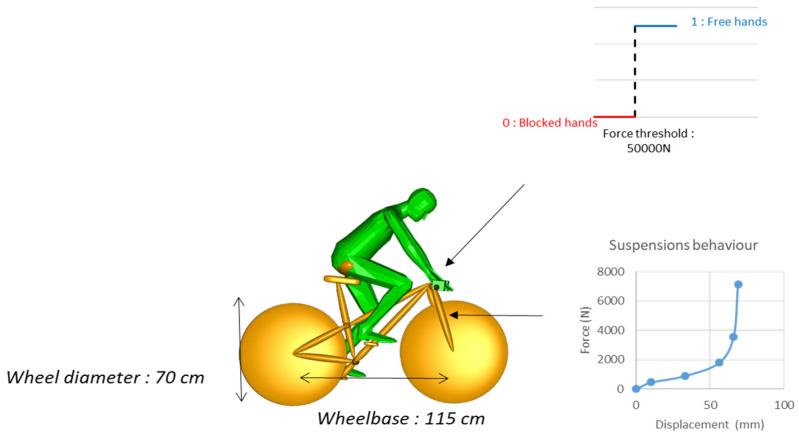
Parameters used and selected for the mountain bike model, including suspension behaviour based on the SRAM [17] design and a handlebar fixation system inspired by Fournier et al. (2023) [18].

**Figure A2:**
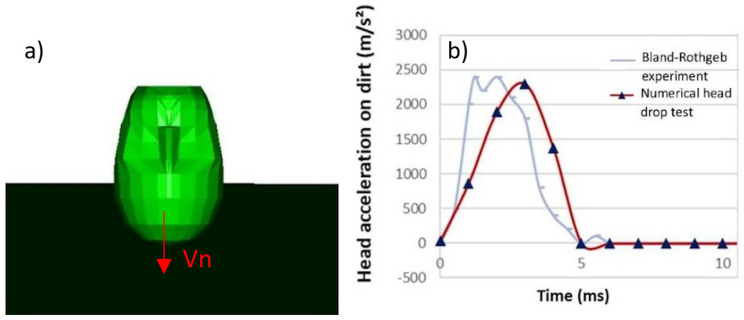
Numerical reproduction of the headform drop test performed at a normal impact speed (Vn) of approximately 4.8 m/s on dirt MTB trails by Bland-Rothgeb et al. [16] (**a**), and head acceleration on dirt over time for the numerical and experimental tests (**b**).

A generic, semi-rigid mountain bike (Decathlon-inspired, medium size) was modelled with a 115 cm wheelbase, 27.5-inch wheels, and a total mass of 12.5 kg. The model incorporates a front suspension fork defined by a sliding joint [17], while the handlebars and wheels were simulated using revolute joints. All characteristics of the mountain bike are described in Appendix A (Figure A1). The rider was positioned in a seated posture on the saddle, with both hands gripping the handlebars (Figure 1E). The rider hands were constrained to the handlebars using a mechanical joint, similar to the approach used by Fournier et al. (2023) [18]. If the force exerted by the hands on the handlebars exceeded a predefined threshold (50,000 N), the rider released the handlebars. Friction coefficients (μ) were applied based on values from the literature: 0.5 between the wheels and dirt ground, 0.3 between the bike and the ground, 0.3 between the rider and the bike, and 0.6 between the rider and the ground [19]. To account for the damping properties of the trail soil, the model’s ground was calibrated based on a previously published experimental head drop test on an MTB trail [16]. The drop test was numerically reproduced, and the damping function was adjusted to match the experimental head acceleration curve, achieving an error of 0.33% on the maximal head acceleration value (Figure 1F and Appendix A, Figure A2).

### 2.3. Global Kinematics Verification of the Rider–Bike Coupling

**Figure 2:**
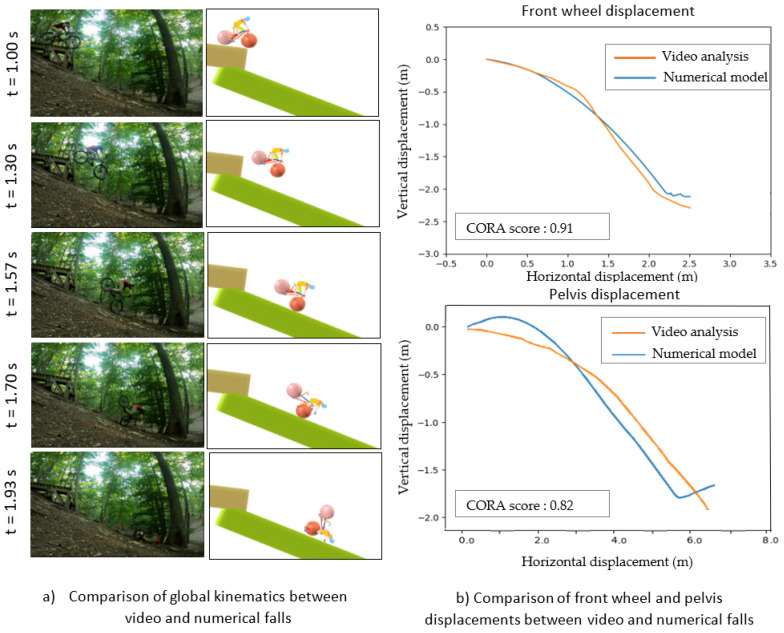
Comparison of numerical and video or experimental falls: (**a**) global kinematics and (**b**) front wheel and pelvis displacements.

To ensure realistic bike–rider interaction during a forward fall, a global kinematic verification was performed. Rather than reassessing the validated human model, this step focused on ensuring that the bike behaviour and resulting rider trajectory were consistent with a realistic over-the-bars (OTB) crash. An in-field OTB crash resulting from a failed jump landing, captured on video, was reconstructed [20] (Figure 2a). The bike and rider’s kinematics were analysed using Kinovea software (Version 0.9.5, Joan Charmant & Contributors, Bordeaux, France, OpenSource software, www.kinovea.org). Two reference points were tracked: the front wheel to assess bike kinematics, and the rider’s pelvis as a proxy for trunk motion (Figure 2b). Assuming the fall occurred in a plane perpendicular to the camera, each frame was scaled based on standard MTB wheel dimensions. The extracted initial conditions—initial slope (13°), landing slope (20°), drop height (120 cm), and riding speed (6.35 m/s)—were used to numerically reproduce the event.

To align the simulation with the video analysis, two key parameters defining the generic bike’s tire mechanical behaviour were calibrated: the damping coefficient (1500 kg/s) and the stiffness (5000 N/mm). These specific parameters were selected as they vary significantly between bicycles and dictate the bike’s macroscopic behaviour during a heavy front-wheel ground impact. Additionally, the properties of the hand-to-handlebars contact were adjusted. This targeted calibration minimized discrepancies, resulting in a close match for fall duration (0.43 s numerical vs. 0.40 s video) and length (2.45 m vs. 2.47 m). The trajectory fit was evaluated using the CORA (CORrelation and Analysis) score, a validated objective rating method used to quantify the degree of correlation between experimental and numerical time-dependent signals [21]. The model achieved a score of 0.91 for the front wheel and 0.82 for the pelvis. Both scores exceed the 0.80 threshold, which is widely accepted in biomechanics as a benchmark for high-fidelity kinematic validation [22]. This preliminary validation (based on a single reconstructed case) confirms that the coupled model can successfully replicate the overall kinematic behaviour of a real-world crash (Figure 2).

### 2.4. Parametric Study and Variables of Interest

To investigate the impact conditions and identify risk factors associated with forward falls, an experimental design was developed to simulate two main causes of a crash leading to forward falls [9].

**Figure 3:**
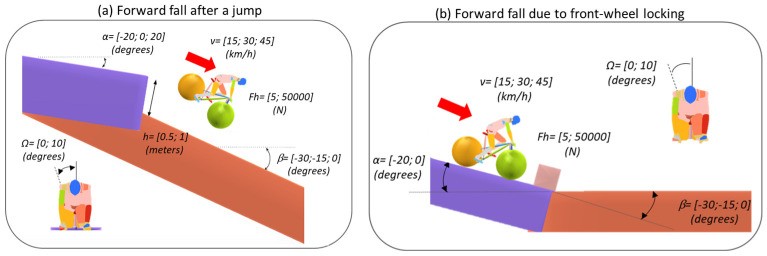
Simulated crash causes: forward falls after a jump (**a**) and forward falls after front-wheel locking (**b**). Both causes feature riding speeds (v) of 15, 30, and 45 km/h (4.2, 8.33 and 12.5 m/s), bike orientations (Ω) of 0° and 10°, final slopes (β) of −30°, −15°, and 0°, and holding forces (Fh) ranging from 5 N (low) to 50,000 N (high). In fall after a jump (**a**), the initial slope (α) is −20° (descent), 0° or 20° (gathered as flat/hill), with fall heights (h) of 0.5 m or 1 m, while in fall after front-wheel locking (**b**), the initial slope (α) is either −15° (descent) or 0° (flat/hill).

1. Forward fall after a jump (Figure 3a): The rider was placed on the initial slope in front of a vertical drop and a landing slope; the forward fall was induced by the bike landing on the front wheel.
2. Forward fall induced by front-wheel locking (Figure 3b): The rider was placed on the initial slope in front of an obstacle simulating either an obstacle on the trail or an excessive braking on the front wheel, inducing a forward fall.

The effect of the following parameters on the fall kinematics and on impact severity indicators was evaluated: the initial slope (α), the landing slope (β), the drop height (h), the initial riding speed (v), the initial bike orientation (Ω) and the handlebars holding force (Fh). The variation levels of the parameters are presented in Figure 3. The riding speed was based on previous studies [23], which reported typical speeds of 20–25 km/h (5.6–6.9 m/s) on downhill trails, with peaks reaching 40–45 km/h (11.1–12.5 m/s). Bike orientation angles were chosen to simulate straight-line riding (0°) or negotiating a bend (10°). Drop heights of 0.5 m and 1 m were selected to accurately reflect the dimensions of intermediate to advanced Technical Trail Features, such as rock drops or jumps, as defined by the International Mountain Bicycling Association (IMBA) trail building standards [24]. The two levels of handlebars holding force correspond either to the release of the handlebars upon the bike’s first impact with the ground after a jump or with the obstacle, or to maintaining a firm grip on the handlebars throughout the entire fall.

The full factorial design of experiment was conducted using HyperStudy (Version 2022.2, Altair Engineering Inc., Troy, MI, USA). Simulations ran with a time step of 1 × 10^−3^ s. A total of 288 configurations were simulated with 216 cases of fall after a jump and 72 of fall after front-wheel locking.

### 2.5. Classification of Impact Kinematics

A recent study by the authors [9] analysed 534 MTB traumatic crashes and classified impact kinematics after a forward fall into four categories:

- Tumbling impact: Head-first impact followed by back impact.
- Locked head impact: Head-first impact with the head remaining locked against the thorax, causing severe neck hyperflexion.
- Head–thorax impact: Head-first impact followed by thorax impact.
- Overflip back impact: Back-first impact following a full airborne rotation.

These impact kinematics categories (further detailed in Section 3) were used to analyse the numerical crash reconstructions. To do this, the order of contact between each body segment and the ground was recorded and used to classify each crash simulation accordingly.

### 2.6. Impact Conditions

The primary focus of this study was extracting the trunk’s global impact conditions, considering the thorax for head–thorax impacts and the back for other types of impact, to define loading environments for future equipment testing. The variables computed were impact tangential, normal and rotational velocities, maximal tangential and normal forces and maximal impact pseudo-energy. Impact speeds and forces were defined as resultant, tangential and normal speeds in the ground coordinate system. The contact between the trunk (chest, abdomen and front pelvis) and the handlebars was also examined (resultant impact force and velocity). The angular velocity of the trunk at impact was computed at the T7 vertebra level considering only the main rotational component (ω_y_). Finally, the impact pseudo-energy (later referred as impact energy) was defined as the work done by the impact force between the trunk and the ground during the first impact of the trunk. The impact pseudo-energy was estimated using Equation (1), where F_r_ represents the resultant impact force of the trunk on the ground, and v_r_ the resultant impact velocity of the trunk, reflecting the energy transferred during the impact.

(1) $$E_{max}=\left(\int_{t_{i}}^{t_{i+1}}F_{r}\left(t\right)\times v_{r}\left(t\right)dt\right),with\;F_{r}\left(t\right)\neq 0\;for\;each\left[t_{i},t_{i+1}\right]$$

Additionally, head impact conditions were considered by computing resultant, tangential and normal impact velocities and forces on ground.

### 2.7. Impact Severity Indicators

While multibody (MB) models lack the localized tissue resolution of finite elements (FE) models for precise injury prediction, established biomechanical metrics were computed as relative severity indicators. These indicators serve to highlight and compare the most critical crash configurations and loading environments, rather than to provide definitive clinical diagnoses.

For the head, the focus was made on the Head Injury Criteria (HIC) (Equation (2)) with an injury threshold of 1000 [25].

(2) $$HIC=max\left({\left(\frac{1}{t_{2}-t_{1}}\int_{t_{1}}^{t_{2}}a\left(t\right)dt\right)}^{2.5}\times (t_{2}-t_{1})\right),$$

where a is the head acceleration and the timestep considered is 36 ms.

For the thoracic and lumbar spine, maximal vertebral forces and moments of each vertebra were compared to thresholds found in the literature (Table 1). A normalized score was computed for both, indicating a rupture risk when the score exceeds 1 (Equation (3)).

(3) $$NVF=\frac{Vertebral\;force}{Force\;rupture\;threshold}\;and\;NVM=\frac{Vertebral\;moment}{Moment\;rupture\;threshold}$$

### 2.8. Statistical Analysis

The effects of initial conditions (fall after a jump vs. fall after front-wheel locking, riding speed, bike orientation, handlebars holding force, initial slope, and final slope) and crash classification (OTB vs. OnTB and impact kinematics) on key impact outcomes, including impact velocities (tangential, normal, and rotational), maximal forces observed (normal and tangential), and the trunk’s maximum impact energy, were analysed using a general linear model. For all explanatory factors (initial conditions and crash classification), the reference categories were 15 km/h (4.2 m/s) for the riding speed, fall after a jump for cause, low for handlebars holding force, descent (−20° vs. flat/hill for +20°/0°) for initial slope, 0° for bike orientation, 0° for final slope, OTB for crash and overflip back impact for cause of the impact kinematics (Table 2 and Appendix C, Table A1). The effects of initial conditions and crash classification on impact severity indicators at various levels (head using HIC, T1–T6, T7–T12, L1–L3, and L4–L5) were evaluated using an ANOVA after assessing residual normality and homoscedasticity with the Shapiro–Wilk and Levene’s tests, respectively. For each quantitative variable and group of parameters (initial conditions and crash classification), model coefficients and *p*-values were reported, with statistical significance thresholds set at 0.05 (*) and 0.001 (**). Results were post-processed with Python (Version 3.10, Python Software Foundation, Wilmington, DE, USA) and Rstudio version 2024.12.1 (Rstudio, Inc., Boston, MA, USA).

## 3. Results

### 3.1. Impact Conditions and Kinematics

**Figure 4:**
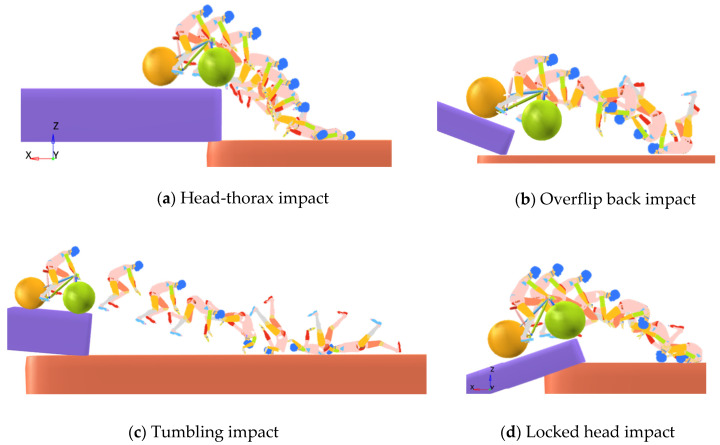
Different impact kinematics while MTB and experiencing a forward fall: Head–thorax impact (**a**); overflip back impact (**b**); tumbling impact (**c**); and locked head impact (**d**). For visibility reasons, the bike was erased after the first image of the fall.

A total of 288 MTB crashes were simulated. Among those, 271 resulted in a forward fall, while the remaining 17 low-kinematics sideways falls were excluded from the analysis. The forward falls were classified into two types of crash: “on-the-bars” (OnTB, 41.7%), when the trunk (chest, abdomen, or front pelvis) of the model impacted the handlebars, and “over-the-bars” (OTB, 58.3%), when it did not (Figure 4). In OnTB scenarios, trunk–handlebars contact occurred mainly at the abdomen (54%) or front pelvis (40%) and rarely at the chest (6%). The median thorax resultant impact speed was 12.47 m/s, and the median peak resultant impact force was 790 N (interquartile range (IQR) 463–1379 N).

**Figure 5:**
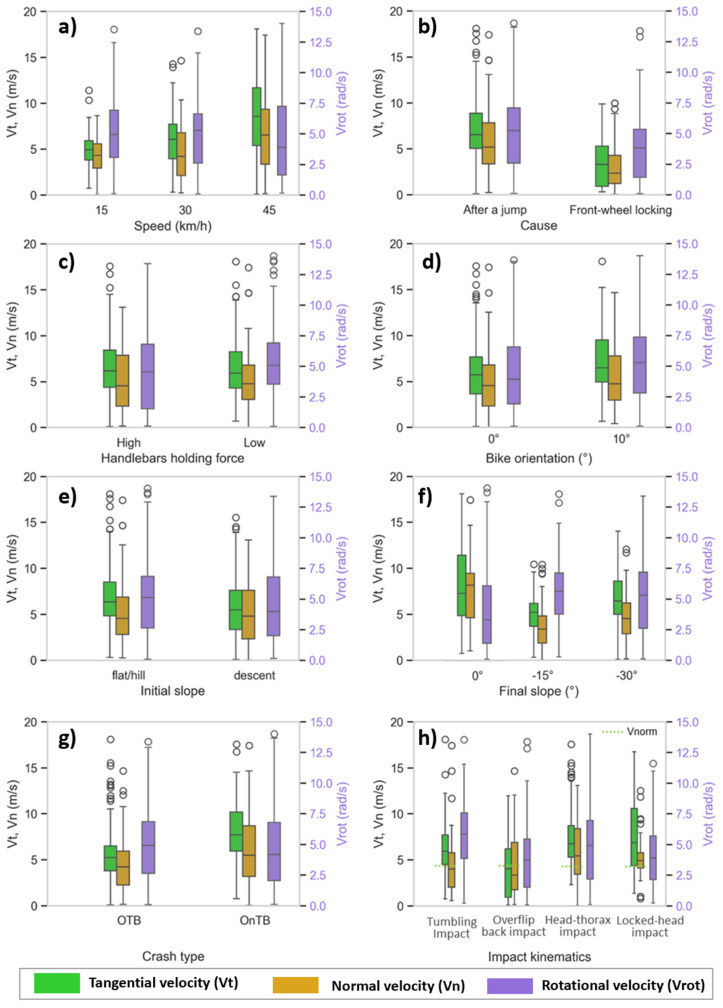
Effects of initial parameters (riding speed (**a**), cause (**b**), handlebars holding force (**c**), bike orientation (**d**), initial slope (**e**) and final slope (**f**)), crash classification (first impact over-the-bar (OTB) vs. first impact on the handlebars (OnTB) (**g**), and impact kinematics (**h**) on tangential velocity (Vt), normal velocity (Vn), and rotational velocity (Vr) of the trunk. Vnorm (4.47 m/s) corresponds to the normal impact speed used in the motorcyclists’ standards EN 1621-2 for back protectors design [8].

**Figure 6:**
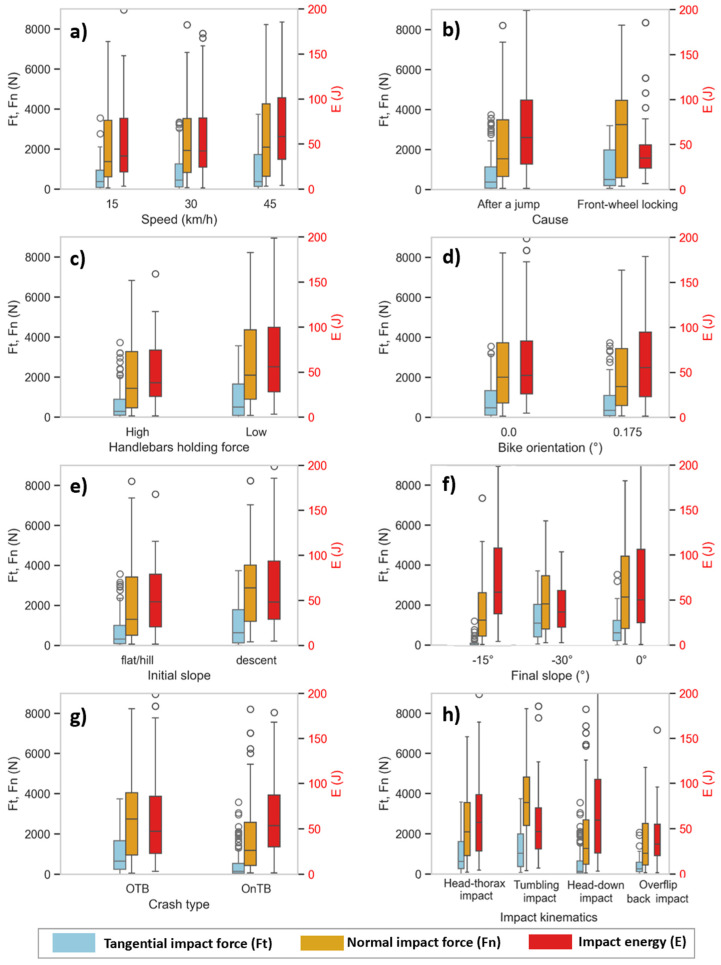
Effects of initial parameters (riding speed (**a**), cause (**b**), handlebars holding force (**c**), bike orientation (**d**), initial slope (**e**) and final slope (**f**)) and crash classification (first impact Over the bar (OTB) vs. first impact On the handlebars (OnTB) (**g**), and impact kinematics (**h**)) on tangential impact force (Ft) and normal impact force (Ft) of the trunk and maximal impact energy (E) of the trunk.

**Figure A3:**
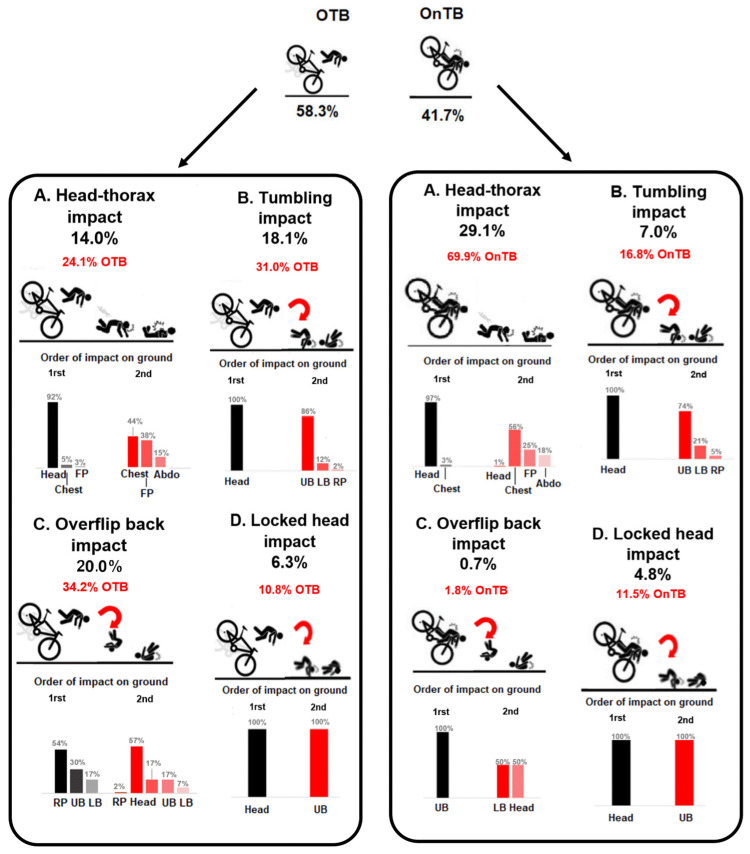
Distribution of cases classified into OTB and OnTB crashes and impact kinematics (tumbling impact, locked head impact, overflip back impact, head–thorax impact), along with the first impacted zones on the ground (head, upper back [UB], lower back [LB], rear pelvis [RP], chest, abdomen [abdo], and front pelvis [FP]). Black bar plots correspond to the first impact area and red bar plots to the second impact area.

**Figure A4:**
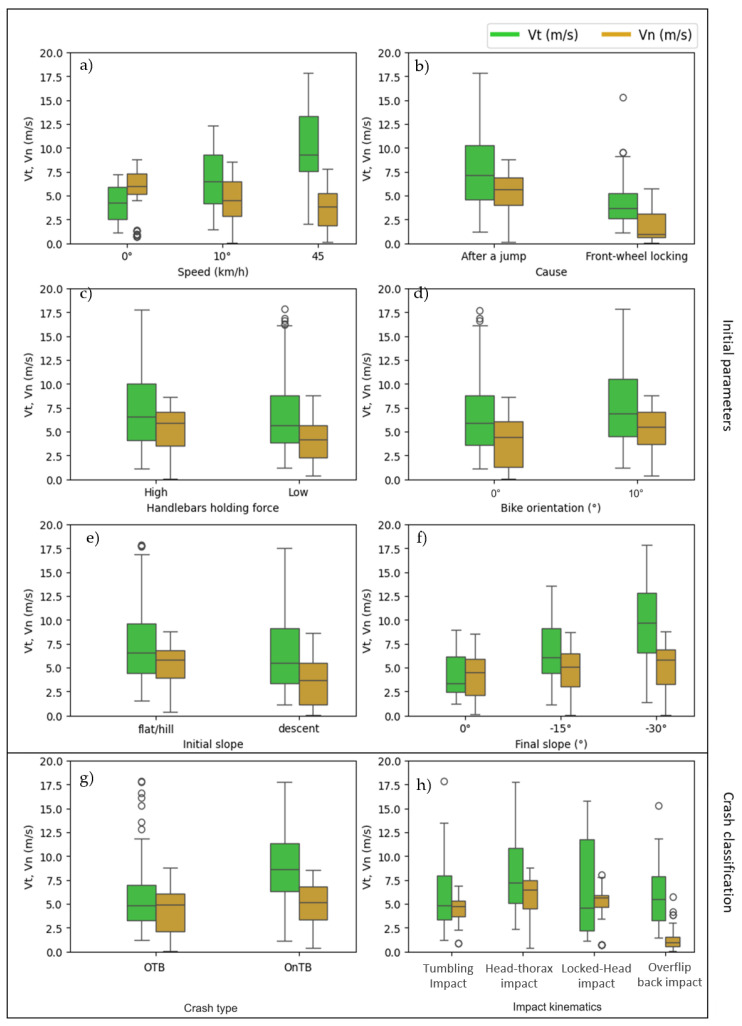
Effects of the initial parameters (riding speed (**a**), cause (**b**), handlebars holding force (**c**), bike orientation (**d**), initial slope (**e**) and final slope (**f**)) and crash characteristics (scenario OTB vs. OnTB (**g**) and impact kinematics (**h**)) on impact velocities of the head (tangential, normal).

**Figure A5:**
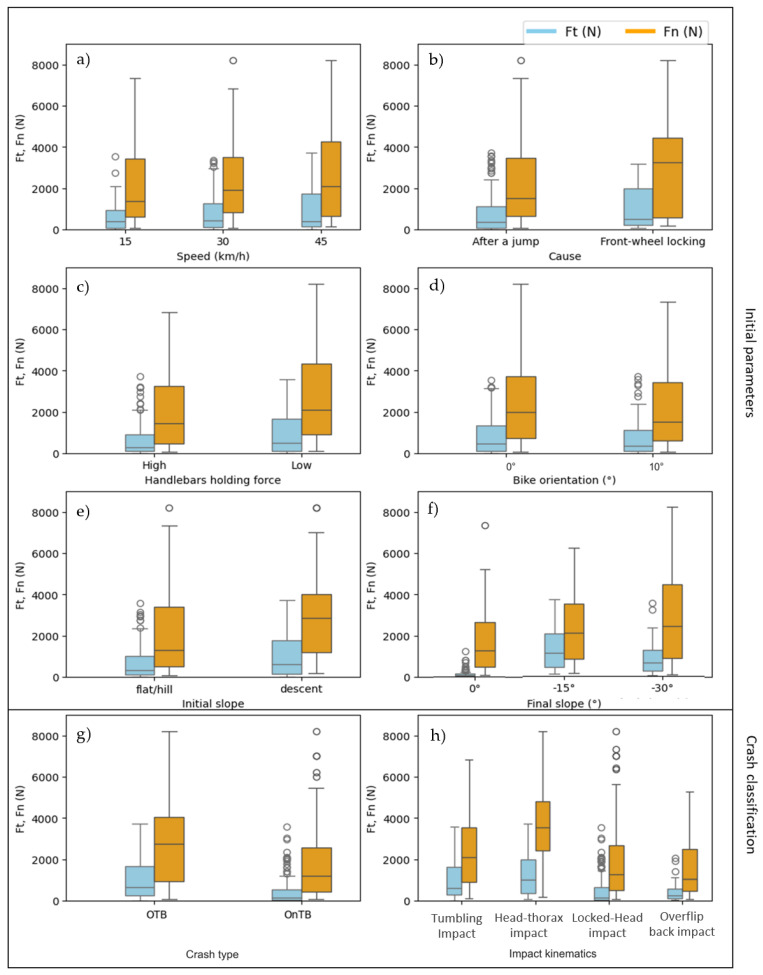
Effects of the initial parameters (riding speed (**a**), cause (**b**), handlebars holding force (**c**), bike orientation (**d**), initial slope (**e**) and final slope (**f**)) and crash characteristics (scenario OTB vs. OnTB (**g**) and impact kinematics (**h**)) on impact forces of the head (tangential, normal).

The impact kinematics on ground were classified into four categories (Figure 4) based on the sequence of impacted body parts, further detailed in Appendix B, Figure A3:

- Head–thorax impact (43.5%) (Figure 4a): The rider fell forward, striking their head first, followed by the thorax. In some cases (2.0%), the thorax impacted first, with the head hitting as a secondary or tertiary contact. The median tangential and normal head impact speeds were 7.22 m/s and 6.48 m/s, respectively (Appendix D, Figure A4 and Figure A5). The thorax primarily hit the chest (52%), followed by the abdomen (29%), with median tangential and normal impact speeds of 6.74 m/s (IQR: 5.29–8.71 m/s) and 5.42 m/s (IQR: 3.43–8.37 m/s) and a median impact energy of 59 J (IQR: 23.0–104.23 J) (Figure 5h and Figure 6h).
- Tumbling impact (25.1%) (Figure 4b): The rider fell head-first (median Vn: 4.77 m/s, Vt: 4.83 m/s) (Appendix D, Figure A4 and Figure A5), and the momentum from speed and slope induced rolling or tumbling. This resulted in a second significant trunk impact, typically on the upper back (83%), lower back (15%), or rear pelvis (3%). At impact median, the tangential and normal speeds of the thorax were 5.94 m/s (IQR: 4.50–7.70 m/s) and 3.98 m/s (IQR: 2.02–5.76 m/s), with a large rotational velocity of 5.85 rad/s (IQR: 3.86–7.57 rad/s) associated with a median impact energy of 57 J (IQR: 25–88 J) (Figure 5h and Figure 6h).
- Overflip back impact (20.7%) (Figure 4c): The rider completed a full airborne rotation, landing directly on their rear pelvis (55%), upper back (32%), or lower back (16%). This mid-air flip concentrated the impact force on the back, making it the primary contact point. The median tangential and normal impact speeds of the trunk were 4.02 m/s (IQR: 0.93–6.17 m/s) and 3.35 m/s (IQR: 1.72–6.88 m/s), with a rotational velocity of 3.73 rad/s (IQR: 1.52–5.44 rad/s). Due to this landing configuration, the back experienced the greatest median impact forces (Fn: 3537 N, Ft: 1020 N) and a median impact energy of 47J (IQR: 28–73 J). Head impacts, if they occurred, were minor (Median Vn of 0.98 m/s) (Figure 5h and Figure 6h).
- Locked head impact (11.1%) (Figure 4d): The rider suffered a direct, forceful head-first impact, locking their body in a head-down position. The neck underwent hyperflexion due to the severe downward force, while the rest of the body remained largely stationary. A secondary impact occurred at the upper back. The median tangential and normal head impacts were 4.61 m/s, and 5.64 m/s (Appendix D, Figure A4 and Figure A5), while those of the upper back were 6.86 m/s (IQR: 4.34–10.58 m/s) and 4.9 m/s (IQR: 4.08–5.76 m/s), respectively (Figure 5h). The median trunk impact energy is 33 J (IQR: 20–55 J).

Among the initial parameters, the primary factors influencing tangential and normal trunk impact velocities were the initial riding speed (Figure 5a), causes (after a jump vs. front-wheel locking) (Figure 5b), and the final slope (Figure 5f). Greater riding speeds were significantly associated with increased normal and tangential impact velocities (*p* < 0.001). Similarly, steep landing slopes and falls after a jump contributed to larger trunk impact velocities (*p* < 0.05) (Table 2 GLM n°1). In falls after a jump, increasing drop height leads to smaller tangential and normal impact velocities but larger trunk impact energy.

Regarding crash classification, OnTB crashes tended to result in greater impact velocities compared to OTB crashes (Table 2 GLM n°2). The type of impact on the ground also significantly influenced tangential impact speed, with head-first impacts (head–thorax impact, locked-head, and tumbling) leading to greater speeds than overflip back impacts. Additionally, trunk rotational impact speed was primarily affected by the landing slope and impact kinematics. It was significantly reduced on steep descents (*p* < 0.001) compared to flat or hilly slopes and increases during tumbling impacts compared to overflip back-first impacts (*p* < 0.05) (Table 2). The maximal impact energy of the trunk was mainly influenced by crash (OTB vs. OnTB) and final slope, with front-wheel locking associated with a reduction in energy (*p* < 0.05) and steeper slopes linked to an increase (*p* < 0.05) (Figure 6 and Table 2).

### 3.2. Impact Severity Indicators

**Figure 7:**
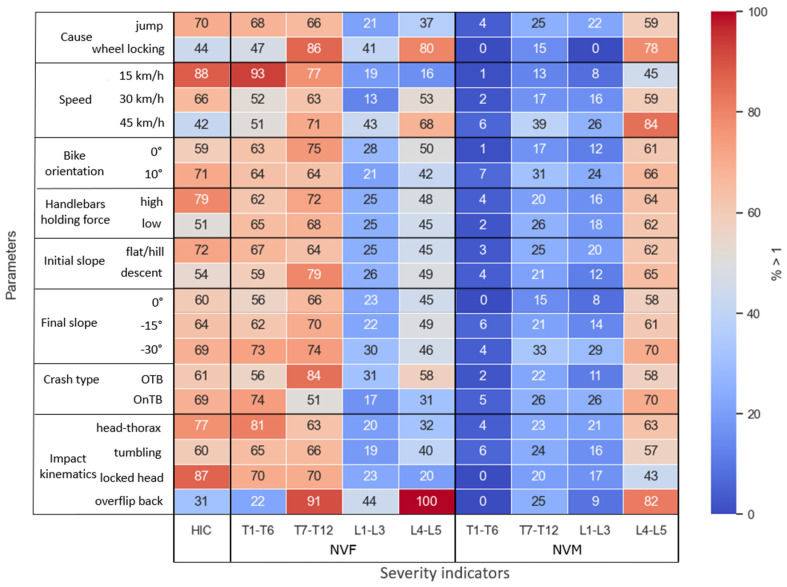
Heatmap of the percentage of cases above the threshold exceedance of three different severity indicators (Head Injury Criteria (HIC), Normalized Vertebral Force (NVF), Normalized Vertebral Moment (NVM)), for different areas (head, T1–T6, T7–T12, L1–L3 and L4–L5), for different initial parameters (bike orientation, handlebars holding force, riding speed, causes, initial slope, final slope) and crash classification (first impact over-the-bar (OTB) vs. first impact on the handlebars (OnTB), impact kinematics).

The Head Injury Criterion (HIC) analysis revealed a median value of 1902 (IQR 599–6395), exceeding the injury threshold of 1000. The HIC was significantly influenced by impact kinematics, cause (front-wheel locking vs. after a jump), handlebars holding force, and riding speed, with a strong statistical correlation (Appendix C, Table A1, ANOVA n°1, *p* < 0.001). Figure 7 illustrates that small riding speeds, forward falls after a jump, and small handlebars forces were associated with an elevated HIC. In contrast, overflip back impacts—where the head does not make initial contact with the ground—show the lowest HIC (31% of cases).

According to the Normalized Vertebral Force (NVF) criterion, high-severity spinal loading, exceeding thresholds, was observed in 89.7% of the simulated cases. The probability of exceeding these thresholds was generally greater at the thoracic level (86.0%) than the lumbar level (49.4%) (Figure 7). The main influencing factors of the NVF were causes (front-wheel locking vs. after a jump), riding speed, crash type (OTB vs. OnTB) and impact kinematics (Appendix C, Table A1). Specifically, front-wheel locking and large riding speed were associated with increased NVF at the thoracic level but smaller NVF at the lumbar level compared to “fall after a jump” and small riding speeds. OnTB scenarios generally posed a greater risk of exceeding NVF thresholds than OTB scenarios, except at the lower thoracic level (T7–T12). Finally, overflip back impact stood out, showing a small NVF at T1–T6 but larger NVF values elsewhere.

The Normalized Vertebral Moment (NVM) criterion indicated a smaller probability of exceeding structural limits than the NVF, especially in the thoracic region (24.4% vs. 86.0%; Figure 7). Unlike the NVF, it showed reduced moment-based risk from T7 to L3 during “front-wheel locking.” Large speeds raised the NVM along the whole spine, and—consistent with NVF—the lower lumbar vertebrae (L4–L5) showed a greater NVM in overflip-back impacts (*p* < 0.001; Appendix C, Table A1, ANOVA n°2).

## 4. Discussion

Mountain biking (MTB) is a rapidly growing sport associated with a relatively large injury rate, particularly in severe cases involving the trunk (spine, chest, abdomen) [1,2,5]. Despite the development of protective equipment such as back protectors, their effectiveness remains unclear due to a lack of epidemiological evidence and the use of non-specific testing standards derived from motorcycling [6,7,8]. This highlights the need for a better understanding of MTB-specific crash mechanisms to define appropriate testing conditions for protective equipment. In this context, the aim of this study was to characterize trunk impact conditions during MTB forward falls, which are a major cause of severe trunk injuries. A multibody model for MTB accident reconstruction was developed, and a preliminary validation was performed by comparing its kinematics with video footage of a single real-world crash. The model was then used to analyse forward MTB crashes. The resulting simulations allowed the classification of four distinct ground impact kinematics: head–thorax impact, tumbling impact, locked head impact and overflip back impact. In most cases (87.1%), the rider struck the ground head first, leading to a probability of exceeding severity thresholds for the head and upper thoracic spine. Focusing on trunk impact conditions, the median normal velocity was 4.61 m/s, and the median impact energy reached 48 J, both close to the 4.47 m/s and 50 J thresholds currently used in standardized testing [8]. Impact values showed notable variability, with median normal velocities ranging from 3.35 to 5.42 m/s and energies from 33 to 59 J depending on the impact kinematics. Furthermore, the presence of substantial tangential (median: 5.97 m/s) and rotational (median: 4.90 rad/s) components challenges the adequacy of relying solely on vertical drop tests to assess protective performance. These findings highlight the complexity of MTB crash scenarios and their influence on trunk impact conditions, offering critical insights for the development and evaluation of mountain biking protective equipment.

### 4.1. Trunk Ground Impact Conditions and Severity

This study presents the first detailed classification of body impacts with the ground during mountain bike crashes, highlighting the influence of impact dynamics on severity indicators. Head–thorax impacts were the most frequent (43.5%), with large impact velocities (Vn: 5.42 m/s, Vt: 6.74 m/s) and an impact energy of 59 J, emphasizing the need to extend thoracic protection beyond handlebars impacts to include ground impacts as well. Other impact scenarios, such as tumbling impacts (25.1%) and overflip back impacts (20.7%), revealed distinct kinematic patterns, involving significant rotational velocities and concentrated forces on the back.

Initial riding conditions, including riding speed, slope, and obstacle interactions, played a critical role in shaping impact dynamics. High speeds and steep final slopes significantly increased both tangential and normal trunk impact velocities. This is consistent with Romanow et al. (2014) [28] who reported that faster riders were more prone to encounter severe injuries. In contrast, forward falls due to front-wheel locking were associated with lower impact velocities compared to jump scenarios. To reduce crash occurrence and severity, jumps and bike park features could be designed to help riders control their speed by adjusting slope steepness or drop heights, as it has been done in winter sports [29,30]. Another important factor to consider is the influence of rider characteristics, including sex, anthropometry, and riding position. The current model represents a 50th-percentile male, which aligns with the primary demographic in MTB [3,4,11], but does not account for variations in body size, mass distribution, or other anthropometric differences that could affect crash dynamics and severity indicators. Future investigations could explore how these factors, along with riding position—such as a standing posture with legs extended, pedals at mid-height (level position), and hips slightly raised off the saddle—alter crash dynamics and impact outcomes. Finally, while this study focused on forward falls, as they are the main contributors to head and trunk injuries in MTB, other crash scenarios, such as lateral or backward falls, may lead to different back impact conditions and consequently different injury patterns. Expanding the scope to include a broader range of fall directions would provide a more comprehensive understanding of MTB crash-related injuries and the associated trunk impact conditions.

The results for the thoracic and lumbar spine revealed distinct risk profiles. The thoracic region demonstrated greater vulnerability to vertebral forces and moments following a poor jump landing, even at relatively low speeds. In contrast, the lumbar spine was more prone to injury after high-speed front-wheel locking on steep descents, especially at the L4–L5 level. Notably, overflip back impacts consistently posed the greatest risk to the lower lumbar spine (82% for NVM and 100% for NVF at the L4-L5 level), underscoring the critical role of mid-air dynamics in spinal injuries leading to rear pelvis or back-first impacts. While Dodwell et al. (2010) [12] reported that thoracolumbar injuries accounted for 26.2% of all spinal injuries, their study did not differentiate between thoracic and lumbar subregions. Our findings provide novel insights into the specific distribution and mechanisms of injury within the thoracolumbar segment. However, it is important to interpret these results with caution, as the current multi-body model is not specifically designed for injury prediction. Multi-body simulations help analyse impact kinematics and impact dynamics but oversimplify human anatomy, limiting their ability to assess localized injury mechanisms, especially in complex structures like the spine. Moreover, injury thresholds selected to compute NVF and NVM scores [26,27,31,32], though widely used, are derived from cadaveric quasi-static compression tests on a single vertebra, which can lead to reduced rupture thresholds compared to multi-vertebra levels at large compressive rate, which is probably more representative to dynamic spinal injury mechanisms.

Finite element (FE) modelling, such as the THUMS spine model, offers a more detailed injury risk assessment by incorporating anatomical structures, material properties, and soft tissue interactions. Integrating FE modelling with multi-body simulations could enhance injury predictions and improve understanding of spinal trauma [33].

### 4.2. Implication for Protective Equipment

Currently, back protectors are designed according to motorcyclist standards [8], requiring them to withstand a 50 J direct normal impact. In this study, a pseudo-impact energy was derived from force–velocity integration during body–ground contact. Although not directly equivalent to the energy used in standard tests, it provides a useful reference for comparison with the 50 J normal impact. The median impact pseudo-energy involved in realistic overflip back impacts was 47J, not far from the values of the standards. Nevertheless, these impacts also exhibited large median rotational velocities (3.73 rad/s) and significant tangential components (4.02 m/s), even greater than normal impact velocities (3.35 m/s). Other impact scenarios involving a secondary back impact, such as tumbling crashes (24.4%), showed a median pseudo-energy transferred to the back (57 J) greater than that of the standard and a high tangential velocities (5.94 m/s). This large tangential speed component was also observed in a study on snowboarding backward falls, where the normal component was on average three times smaller than the tangential component (Vn: 2.4 m/s vs. Vt: 7.3 m/s) [15]. These results suggest that the current evaluation and design approach of back protectors, which considers only normal impact forces, might overlook rotational and tangential components that may also contribute to vertebral injuries in MTB crashes.

### 4.3. Other Findings of the Study: Head, Cervical Spine, and Handlebar Impact Kinematics

While, the main focus of this study was the impact of the trunk against the ground, several other interesting impacts were observed. First, 42.7% of simulated crashes involved a high-energy impact between the trunk and the handlebars prior to ground contact. Such impacts can result in severe injuries, including isolated spleen or liver ruptures [34,35,36]. Although these injuries are more frequently reported in children [34,37] while cycling, they also occur in adults and often require surgical intervention [38]. This suggests that impacts between the trunk and the handlebars should be considered in the design of mountain biking thoracic protection, and that the reported impact resultant velocities (median: 12.47 m/s) and forces (median: 790 N) provide important insights for improving the design of such protective equipment.

Second, in 87.1% of the simulations, the head was the first body region to impact the ground, resulting in HIC values exceeding its threshold in 64.6% of cases and indicating a potential risk of cervical injury. This is consistent with Dodwell et al. [12] who reported that over 70% of severe spinal injuries occurred in “over-the-bars” incidents with head-first impacts. In this study, the cervical spine was the most frequently injured spinal region (73.8% of spinal injuries [12]), which can result in neurological complications, emphasizing the critical risks associated with head-first impacts. Although HIC values exceeded its threshold in most of the simulated cases, it is likely that this model overestimated head injury risk, as it does not account for muscle activity, protective reflexes, or the presence of a helmet. Indeed, head or brain injuries represent a quarter of all injuries [39] and can be up to 57.1% for severe cases (Injury Severity Score above 12) [29]. Indeed, the existing literature suggests that helmet use reduces the risk of head and brain injuries by 63–88% and helmet-wearing rates among mountain bikers range from 75 to 100% [4,12]. The head impact velocities from our simulations offer valuable insights for helmet testing. Current MTB helmet standards, adapted from general cycling practices, assess normal head impacts at 5.42 m/s [40]. Our simulations showed normal velocities within this testing range with a median value of 5.05 m/s, with peaks reaching 6.48 m/s in head–thorax impacts. However, tangential velocities were notably greater, with a median of 6.44 m/s. These values differ slightly from those reported by Bourdet et al. (2012) [41] in their multi-body modelling of road cycling crashes, which showed a mean normal velocity of 5.2 m/s and a tangential velocity of 3.7 m/s. This discrepancy likely reflects the unique crash dynamics involved in mountain biking. Given the significant role of tangential velocity in generating rotational accelerations linked to brain injury risk, our findings may suggest considering the specificity of these complex and high-energy impacts when designing and evaluating a mountain biking helmet.

### 4.4. Limits

While this study provides novel insights into MTB crash dynamics, several limitations must be acknowledged. First, the model was verified in three stages: (1) human body response was previously validated using reconstructions of cadaveric and ATD pedestrian and snowboarding impacts, (2) ground response was calibrated through reconstructions of ATD head impacts, and (3) overall kinematics of the MTB/rider model were verified using the reconstruction of a real-world mountain biking forward fall captured on video. However, this final verification relied on a single case, which cannot represent the full variability of crash kinematics, and was limited to kinematic comparison only, without kinetic measurements such as ground or handlebar impact forces. Second, in this work, the effects of several initial crash parameters on impact conditions were investigated, but other parameters that may also strongly influence these conditions were kept constant. In particular, ground damping was calibrated based on impacts on dirt, but could vary depending on the surface (e.g., stiffer for rocks or softer for grass). Similarly, tire and suspension behaviour—which differ between bikes—were fixed. In addition, the crash kinematics were evaluated for a seated rider position, whereas rider posture (e.g., standing, as commonly adopted during descents and jumps) may significantly affect impact conditions. Furthermore, the current model represents only a 50th-percentile male, as males were the primary injured population in previous studies [3,4,39], without accounting for variations in sex, anthropometry, or riding position that may influence crash dynamics and injury risk. Moreover, the analysis is restricted to forward falls, whereas other crash configurations (e.g., lateral or backward falls), though less frequent [10,11], could also result in different trunk impact conditions and injury mechanisms. Further work should therefore investigate these additional parameters and reconstruct additional real-world crashes, as well as conduct controlled experimental reconstructions, to extend the validation of the model and assess the robustness of the presented results. Third, the effects of crash parameters on impact conditions were systematically evaluated using a full factorial design of experiments. Although the selected parameter ranges were realistic, the simulations are, by design, not independent empirical observations, and the reported crash proportions do not represent the likelihood of crash occurrence in the real world. Finally, this study identified a specific spinal severity indicator based on localized force and moment loading. However, the multi-body model was not originally designed for direct clinical injury prediction and may oversimplify human anatomy. Although the spinal response under various loading conditions was validated in a previous study, the estimated severity indicator should be interpreted cautiously as relative indicators of spinal loading and crash severity. Further studies using refined finite element spine models are needed to better characterize the specific injury mechanisms under the impact conditions identified in this work.

### 4.5. Perspectives

The use of advanced multibody simulation in this study offers a valuable methodological contribution to sports medicine by enabling detailed analysis of crash dynamics that are difficult to capture through experimental or epidemiological approaches alone. By replicating realistic crash scenarios, the methodology bridges a critical gap between laboratory testing and real-world injury mechanisms, providing insights of fall trajectories and shedding light on the role of tangential and rotational back impact velocities in spinal injury mechanism. This may question current standard methodologies, which often rely on simplified vertical impacts, and suggests the need for more representative test protocols in helmet and back protector certification. Combined with finite element modelling, this approach may be particularly relevant for understanding complex injury patterns such as traumatic brain injuries and spinal trauma in mountain biking. Similar methods may be applicable in other extreme sports, whose experimental reconstruction might be difficult, to bring insights for injury prevention strategies and design of protective equipment.

## 5. Conclusions

This study provides a comprehensive analysis of fall dynamics and potential injury risks in mountain biking forward crashes using a multi-body model. By classifying impact scenarios and quantifying associated kinematic parameters, we identified critical conditions that may pose large risks for the head and thoracolumbar spine. This is the first study to provide values of critical and realistic impact conditions (velocities, forces, energies) of the trunk during four distinct crash scenarios that can provide relevant boundary conditions to inform the evaluation of current back protectors on the market. The results suggest that MTB-specific back protector testing may need to consider tangential and rotational components, in addition to the currently considered normal impact force. These insights mark an important step toward improving rider safety through better-informed design, testing, and regulation of protective systems, tailored for the unique kinematics of mountain biking.

## Figures and Tables

**Table 1:** Rupture threshold in flexion moment and compression force for each vertebral area from the literature.

Vertebral Areas	Thoracic	Lumbar
Flexion moment rupture threshold (Nm)	277.5 Yoganandan et al. (1988) [26]	277.5 Yoganandan et al. (1988) [26]
Compression force rupture threshold (N)	2953 Yoganandan et al. (1988) [26]	4500 Stemper et al. (2018) [27]

**Table 2:** General linear model for each quantitative impact variable with (1) initial parameters, including riding speed, cause, handlebars holding force, initial slope, bike orientation and final slope, and (2) crash description, including crash type and impact kinematics. * Corresponds to a significance threshold of 0.05 and ** of 0.001.

	Vt (m/s)	Vn (m/s)	Vrot (rad/s)	Ft (N)	Fn (N)	E_max (J)
**GLM n°1**						
Intercept	3.54 **	3.69 **	5.18 **	1257 **	2189 **	46.44 **
Speed: 30 km/h (8.3 m/s)	1.23 **	0.75 *	−0.21	155	150	−8.87
Speed: 45 km/h (12.5 m/s)	4.33 **	2.53 **	−0.28	243 *	507	17.49
Cause: Front-wheel locking	−4.23 **	−3.09 **	−0.89	131	626 *	−34.18 *
Handlebars holding force: High	0.06	−0.13	0.59	269 *	966 **	11.97
Initial slope: Flat/Hill	0.16	−0.74 *	0.12	−315 **	−888 **	−15.39
Bike orientation: 10°	0.25	−0.26	0.63	−28	−183	6.87
Final slope: −15°	1.94 **	1.01 *	−0.17	−505 **	532	34.87 *
Final slope: −30°	3.22 **	3.82 **	−1.33 *	−1231 **	−512	51.39 **
**GLM n°2**						
Intercept	4.12 **	4.57 **	4.23 **	1302 **	3790 **	58.24 **
Crash: OnTB	1.85 **	1.08 *	−0.31	−257 *	−588 *	−3.39
Impact kinematics: Head–thorax impact	2.12 **	0.51	0.99	−616 *	−1461 **	36.75 *
Impact kinematics: Locked head impact	2.45 **	0.17	0.1	−732 *	−1917 **	2.74
Impact kinematics: Tumbling impact	1.61 *	−0.54	1.65 *	−259	−1100 *	14.87

## Data Availability

The data presented in this study are available on request from the corresponding author.

## Abbreviations

The following abbreviations are used in this manuscript:

ATD	anthropomorphic test device
FE	finite element
FP	front pelvis
FSU	Functional Spinal Unit
HIC	Head Injury Criterion
ISS	Injury Severity Score
LB	lower back
MB	multibody
MTB	mountain biking
NVF	Normalized Vertebral Force
NVM	Normalized Vertebral Moment
OnTB	on-the-bars
ROM	range of motion
RP	rear pelvis
TBI	traumatic brain injury
UB	upper back

## Appendix C. Statistical Analysis of Initial Circumstances and Crash Description on Injury Predictions

**Table A1:** F-statistics of the ANOVA tests for different injury criteria of the head and the spine, with (1) initial parameters, including riding speed, cause, handlebars holding force, initial slope, bike orientation and final slope, and (2) crash description, including crash type and impact kinematics.

	HIC	NVF				NVM			
		T1–T6	T7–T12	L1–L3	L4–L5	T1–T6	T7–T12	L1–L3	L4–L5
**ANOVA model n°1**									
Riding speed	13.04 **	5.89 *	11.38 **	9.33 *	33.24 **	3.39	4.60 *	21.31**	5.19 *
Cause	28.42 **	26.45 **	2.18	14.07 **	33.51 **	2.32	10.89 **	5.55 *	17.09 **
Handlebars holding force	32.63 **	0.35	0.42	6 × 10^−5^	0.37	1.10	1.88	0.13	0.04
Initial slope	8.81 *	1.60	2.69	0.25	0.06	1.14	0.05	0.31	0.11
Bike orientation	2.67	0.12	0.92	0.48	0.10	5.73 *	5.49 *	2.36	2.19
Final slope	0.81	3.71 *	0.78	1.06	0.15	2.58	5.06 *	9.10 **	1.70
**ANOVA model n°2**									
Crash (OTB vs. OnTB)	1.98	11.59 **	37.08 **	7.35 *	26.59 **	2.40	0.63	10.61 *	4.18 *
Impact kinematics	17.37 **	20.63 **	1.62	2.79 *	30.45 **	1.25	0.32	0.11	7.79 **

* Corresponds to a significance threshold of 0.05 and ** of 0.001.
